# Lesion complexity drives age related cancer susceptibility in human mammary epithelial cells

**DOI:** 10.18632/aging.101183

**Published:** 2017-02-28

**Authors:** Deepa M. Sridharan, Shiena Enerio, Mark A. LaBarge, Martha M. Stampfer, Janice M. Pluth

**Affiliations:** ^1^ Division of Biological Systems and Engineering, Department of Organismal Systems and Bioresilience, Lawrence Berkeley National Laboratory, Berkeley, CA 94803, USA; ^2^ Department of Population Sciences, City of Hope National Medical Center, Duarte, CA 91010, USA

**Keywords:** age of exposure, breast cancer susceptibility, complex lesions, centrosome aberrations, stem cells, genome instability

## Abstract

Exposures to various DNA damaging agents can deregulate a wide array of critical mechanisms that maintain genome integrity. It is unclear how these processes are impacted by one's age at the time of exposure and the complexity of the DNA lesion. To clarify this, we employed radiation as a tool to generate simple and complex lesions in normal primary human mammary epithelial cells derived from women of various ages. We hypothesized that genomic instability in the progeny of older cells exposed to complex damages will be exacerbated by age-associated deterioration in function and accentuate age-related cancer predisposition. Centrosome aberrations and changes in stem cell numbers were examined to assess cancer susceptibility. Our data show that the frequency of centrosome aberrations proportionately increases with age following complex damage causing exposures. However, a dose-dependent increase in stem cell numbers was independent of both age and the nature of the insult. Phospho-protein signatures provide mechanistic clues to signaling networks implicated in these effects. Together these studies suggest that complex damage can threaten the genome stability of the stem cell population in older people. Propagation of this instability is subject to influence by the microenvironment and will ultimately define cancer risk in the older population.

## INTRODUCTION

DNA damage is believed to be the initial insult that underlies carcinogenesis and the process of aging. In addition to endogenous lesions caused by reactive oxygen species (ROS), cells are subject to a variety of environmental stresses that can damage their DNA. While oxidative radicals can cause simple lesions such as base damages or strand breaks, additional damages occurring in close proximity within the DNA can result in complex lesions that consist of two or more types of DNA damages within a single turn of the helix. The ability to effectively repair all these lesions in an error-free manner influences cancer susceptibility. Interestingly, the consequence of stochastic accumulation of deleterious lesions in post-mitotic cells over the lifetime of an individual also contributes to aging, a life stage characterized by gradual deterioration of function and increased risk of diseases such as cancer [1]. Progressive decline in DNA repair efficiency, increased oxidative burden, telomere shortening and disrupted tissue architecture are all key factors that contribute to transformation in older cells [2, 3]. For example, 80% of the breast cancer patients are diagnosed over the age of 50 [4], suggesting that cumulative damages may be a considerable risk factor for developing breast cancer. Whether the nature of the DNA lesion has an impact on cancer susceptibility in older individuals is unknown. This is especially significant given the current increases in human longevity achieved through medical advances. To center on this question, we have used radiation as a tool to examine age dependent differences in biological response based on the complexity of the damage.

Radiation is a well-known carcinogen that can cause both simple and complex DNA lesions. Environmental exposures range from mGys from high background radiation to 60-80 Gy received during fractionated radiotherapy. Given the dramatic increase in diagnostic and therapeutic radiation exposures in the past few decades it is essential to understand its carcinogenic potential, especially in radiation-sensitive organs such as the human mammary gland. Older individuals, who typically have a higher incidence of cancer also coincidentally, receive higher cumulative exposures. If carcinogenesis were proportional to exposure, then risk would be significantly higher in older women. However epidemiological data from two different radiation-exposed populations, the Japanese who survived the nuclear explosions in 1945 and children who are clinically exposed to radiation, contradict this postulate. They reveal that individuals exposed at an early age to low non-lethal doses of gamma rays, which primarily cause simple damages, exhibit higher excess relative risk of developing radiological cancers [5, 6] (United Nations Scientific Committee on the Effects of Atomic Radiation: 2013 Report). This increased risk for individuals exposed at a young age has been attributed to the availability of a longer post-exposure period for cancer to develop. However, how complexity of the initial lesion impacts down-stream events that inform cancer susceptibility in old vs. young individuals has not been defined. Breast cancer risk appears to decrease with increasing age of radiation exposure not only for A bomb survivors but also for radiotherapy cohorts [7]. The mechanistic underpinnings of these age-related differences in radiation-induced breast cancer susceptibility are not well understood. As different exposures can elicit a range of DNA lesions from simple to complex or a combination of both, it is equally important to understand how the complexity of damage impacts age-related cancer susceptibility. To clarify this, we assessed two different surrogate markers of cancer; namely centrosome amplification and increase in stem cell numbers, to test the hypothesis that the age of an individual can predict cancer predisposition in response to complexity of the lesion induced.

Centrosomes are microtubule-organizing centers that orchestrate a wide variety of cellular processes such as chromosome segregation, cell motility, adhesion, signaling and acquisition of polarity [8]. They are comprised of two centrioles surrounded by pericentriolar matrix. There is overwhelming evidence that suggests that the structure, function and the number of centrosomes are strictly controlled by tightly regulated mechanisms in order to maintain genomic integrity by ensuring mitotic fidelity. Thus it is not surprising that the consequences of centrosome aberrations can be catastrophic. Centrosomes faithfully divide once per cell cycle and maintain genomic integrity by ensuring mitotic fidelity [9-11]. Numerical and structural aberrations in centrosomes have been known to destabilize the genome by promoting aneuploidy and have been implicated in cancer development [12, 13]. Centrosome defects have been noted in nearly all types of human cancers [14] and shown to increase with tumor grade [15, 16]. In the breast, centrosome aberrations have been detected even in premalignant lesions and correlate with genetic instability [17, 18]. However the effect of age on baseline and radiation-induced centrosome aberrations is not known.

Adult stem/progenitor (S/P) cells have the ability to renew and repopulate the cellular niche with mature differentiated cell types specific to the tissue of origin. Thus they play a significant role in regeneration, repair and maintenance of tissue homeostasis. Conversely, mutations that compromise their genetic integrity can potentially give rise to cancer. One subpopulation, coined the “cancer stem cell”, shares attributes of the adult stem cell, but is thought to have tumor initiating properties. While the validity of the cancer stem cell hypothesis as the causative tool for carcinogenesis is still under debate, a growing body of evidence from tumors of different tissue origin, including the breast, strongly supports the existence of a sub-population of stem cells within cancers with tumorigenic potential [19]. Studies have shown that stem cells within the normal adult tissue and cancers have common features. Both share an integral ability to either self-renew and create clonal progeny or initiate terminal differentiation through the stepwise generation of multi-potent and then committed progenitors. This programmed differentiation is however aberrant in cancers and is thought to be the root cause underlying tumor heterogeneity. Interestingly, resurgence of tumors after radiation therapy has been attributed to the ability of this unique population to resist radiation exposure [20-22]. This finding highlights the importance of enumerating S/P populations following radiation exposure, as genomic instability in this population could be propagated through generations and increases the potential for cancer development. An increase in S/P population has been observed following radiation exposures that result primarily in simple damages [20], however the impact of complex lesions on S/P numbers has not been characterized, especially at different ages of exposure. Although cancer prevalence is thought to increase with both age and radiation exposure, whether their effects are additive or synergistic in altering the number of S/P cells, is not known.

In order to understand the combined effects of age and damage complexity on centrosome number and stem cells, we exposed a panel of 15 well-characterized finite-lifespan normal human primary mammary epithelial strains from a wide age-range (young, middle-aged, old) to two types of radiation; Titanium ions (Ti) that primarily generate complex lesions and Cesium (Cs) that predominantly causes simple lesions. These strains were previously reported by Garbe et al. [23]. Cells were cultured and evaluated at long times following radiation exposure. To provide mechanistic clues to these phenotypes we also assessed phosphorylation of key nodes in pathways involved in proliferation and inflammation. Our studies reveal that the two surrogate cellular phenotypes of cancer exhibit a unique relationship with age and lesion complexity. Their combined effects could enhance cancer predisposition in the older population. Phosphorylation signatures provide a glimpse into the mechanistic links that underlie these age related effects specific to lesion complexity.

## RESULTS

### Dose dependent increase in centrosome aberrations

**Figure 1:**
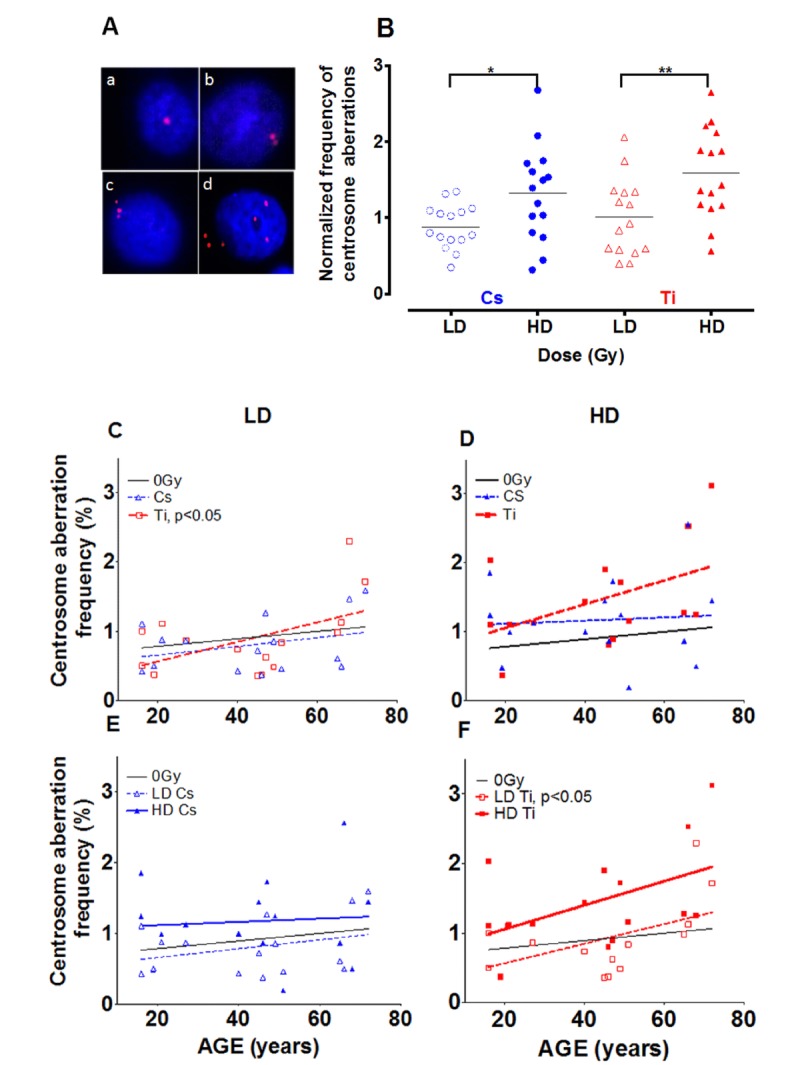
Effect of dose on the frequency of centrosome aberrations Fifteen HMEC strains derived from individuals of various ages were exposed to a low dose (LD) and high dose (HD) of two types of radiation namely Cs and Ti ions. Roughly equitoxic doses of Cs and Ti were used for exposures (LD-CS = 0.12 Gy; HD-CS = 0.8 Gy; LD-Ti = 0.05 Gy; HD-Ti = 0.5 Gy). Aberrant numbers of centrosomes per cell were visualized by indirect immunofluorescence microscopy using an anti-pericentrin antibody (**A**). Staining was carried out within days of fixation, and the number of cells containing aberrant numbers of centrosomes was counted in random regions of the slide. The majority of the cells in irradiated and unirradiated samples show one or two pericentrin foci **(A: a, b)**. Cells with supernumerary centrosomes showed numbers of centrosomes ranging between 3 to as many as 7, and were scored as aberrant (>3P) (**A: c, d**). Cells were processed 9 days post-exposure and the percentage of aberrant cells in each strain was plotted relative to the unexposed population (**B**). Data are based on four independent experiments for high dose and two independent experiments for low dose. The frequency of cells with supernumerary (>3P) centrosomes was graphed against age of the individual from which the strain was derived (**C-F**). Regression analysis was used to model the relationship. The effects of lesion complexity on this relationship at a low (**C**) and high dose (**D**) were graphed. The effect of dose for Cs (**E**) and Ti ion exposure (**F**) were also graphed. Averaged data and the regression line for Cs and Ti ion exposed samples is shown with blue and red, symbols and lines respectively. In Fig. 1E and 1F, LD and HD are distinguished using dotted and full lines, respectively.

Centrosome aberrations were assayed as an indicator of genomic instability and a potential surrogate marker for increased cancer risk. Previous studies have noted an increase in centrosome aberrations following radiation exposure [24-26]. However, the effect of dose and lesion complexity on HMEC strains derived from women of different ages has not been previously defined. Cells in early passage (p4) were exposed to a low and high dose of Cs or Ti 300 MeV/n (herein referred to as Ti) radiation and the frequency of centrosome aberrations was defined for each strain. Centrosomes were visualized using fluorescent microscopy by detecting fluorescently tagged pericentrin, a major component of the pericentriolar matrix surrounding the centrosome. Representative images of cells with normal and aberrant numbers of centrosomes are shown in Fig. 1A. Cells with 1-2 centrosomes were characterized as normal (Fig. 1A: a, b) and >2 were considered aberrant (Fig. 1A: c, d). The frequency of cells containing aberrant centrosomes in each strain was graphed relative to its unexposed baseline (Fig. 1B). The black horizontal line indicates the mean frequency of aberrant cells in the population with each exposure. The relative proportion of aberrant cells did not notably change in cells exposed to low doses of radiation relative to controls (Fig. 1B). In contrast, when exposed to a high dose (HD), a 1.3-fold increase in the frequency of aberrant cells was noted (range 0.3-2.7) for Cs and a 1.6-fold (range 0.56-2.6) for Ti (Fig. 1B). The frequency of centrosome aberrations significantly increased following high doses in comparison to low dose (LD) following both Cs, which creates mainly simple lesions (p<0.05) and for Ti which creates primarily complex lesions (p<0.01).

### Impact of age of exposure on centrosome aberrations

To assess age as a prognostic factor in radiation induced cancer risk, we next investigated the relationship between the age of the individual who provided the mammary tissue and the frequency of aberrations induced using linear regression (Fig. 1C-F). For clarity, low and high doses are distinguished using dotted and full lines respectively and centrosome aberration data from Cs (blue) and Ti (red) exposures are presented. The baseline for centrosome aberrations in unexposed cells shows a slight increase with age that is not significant (black line Fig. 1C-F). The top panel (Fig. 1C and 1D) compares the frequency of aberrations induced by Cs and Ti exposures at low (left) and high doses (right) and reveals that the percentage of cells with supernumerary centrosomes increases linearly with age, particularly when exposed to Ti. The increase in centrosome aberrations after high dose Cs exposure is negligible and appears to be independent of age. Our data suggest that for every year increase in age, the centrosome aberration frequency increases by ~ 0.02 fold for both low and high dose Ti exposure. The linear trend is significant at low dose (p<0.05) where samples over the age of 50 years reveal an aberration frequency significantly higher than control. A comparison of dose effects within each radiation type suggests that the slopes are not significantly different for Cs. For Ti, the slopes for LD and HD appear parallel; with the high dose exposure exhibiting a 1.9-fold increase in centrosome aberrations relative to the low dose, for the same age group (Fig. 1D). As polyploidy is often observed in cells with higher levels of centrosome aberrations, ploidy was assessed following a high dose Ti exposure using flow cytometry analysis of Propidium Iodide (PI) stained samples. Fitting with an increase in centrosome aberrations, an age dependent increase in polyploidy (cells >4C DNA content) was observed with radiation exposure (data not shown).

### Distribution of the number of centrosomes in aberrant cells after radiation exposure

**Figure 2:**
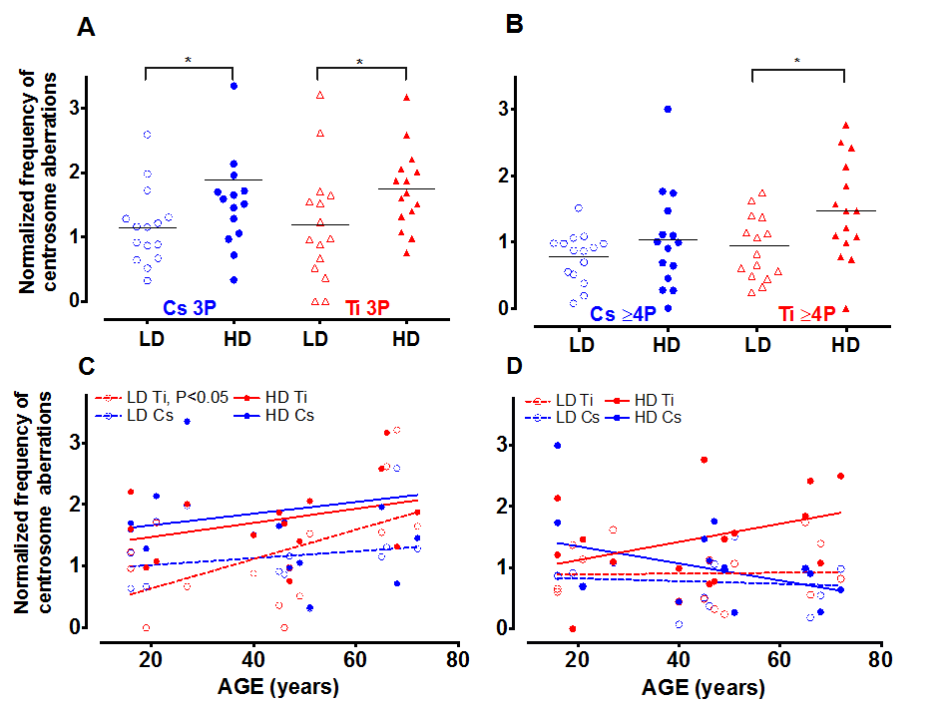
The number of centrosomes per cell varies with dose and age Centrosome aberrations were further classified into cells containing 3 centrosomes (3P= 3 pericentrin foci) and those that had 4 or more (≥4P pericentrin foci). The fraction of cells with 3P and >4P were graphed relative to control. Roughly equitoxic doses of Cs and Ti were used for exposures (LD-CS = 0.12 Gy; HD-CS = 0.8 Gy; LD-Ti = 0.05 Gy; HD-Ti = 0.5 Gy). The dose effect on the change in 3P (**A**) and ≥4P (**B**) populations relative to control is shown. Wilcox test was used to determine statistical significance, *=p<0.05. The normalized proportion of aberrant cells with either 3P (**C**) or >4P (**D**) was plotted as a function of age. Regression lines analyzing the trends as a function of age were fitted to these data.

Next we characterized how the nature of the centrosome aberrations was impacted by simple and complex damages. Aberrant cells (>2 centrosomes) identified while scoring a minimum of 400 cells were classified into two sub-groups; cells with 3 pericentrin foci (3P) and those containing 4 or more pericentrin foci (≥4P, Fig. 2). Representative images for cells with 3P and ≥4P foci/cell are shown in Fig. 1A: c and d, respectively. Cells containing greater numbers of centrosomes per cell (≥5) were noted with very low frequency and hence were pooled with the >4P subgroup. For both radiation types, the 3P subgroup was significantly increased with high dose as compared to cells exposed to low dose (Fig. 2A). The mean increase for 3P was not significantly different between Cs and Ti (Fig. 2A). In comparison, there is a notable increase in the ≥4P subgroup with high dose Ti exposure that is significantly higher than low dose (p<0.05 Fig. 2B). We next employed regression analysis to assess how the frequency of these sub-groups varies with age (Fig. 2C, 2D). The relationship between the number of centrosomes per cell and age revealed interesting trends. The proportion of cells with 3P showed an increasing trend with age for high dose exposure for both radiation types (Fig. 2C). At low doses, the fraction of cells with 3 centrosomes showed a significant linear increase with age (p<0.05) for Ti exposure, but not for Cs exposure (Fig. 2C). However this relationship was not noted for the ≥4P population exposed to low doses of either radiation type (Fig. 2D). In contrast, for high dose exposures, the ≥4P population displayed an age dependent increase for Ti but a decrease with Cs exposure (negative slope). With high dose Cs exposure, the inverse relationship between age and fold change in centrosome aberrations relative to control, appears to be a discrepancy at first glance. However, this age-associated decrease when calculating fold values can be attributed to the higher baseline centrosome aberration frequency in unexposed cells from older people (Sup. Fig. S1).

**Figure 3:**
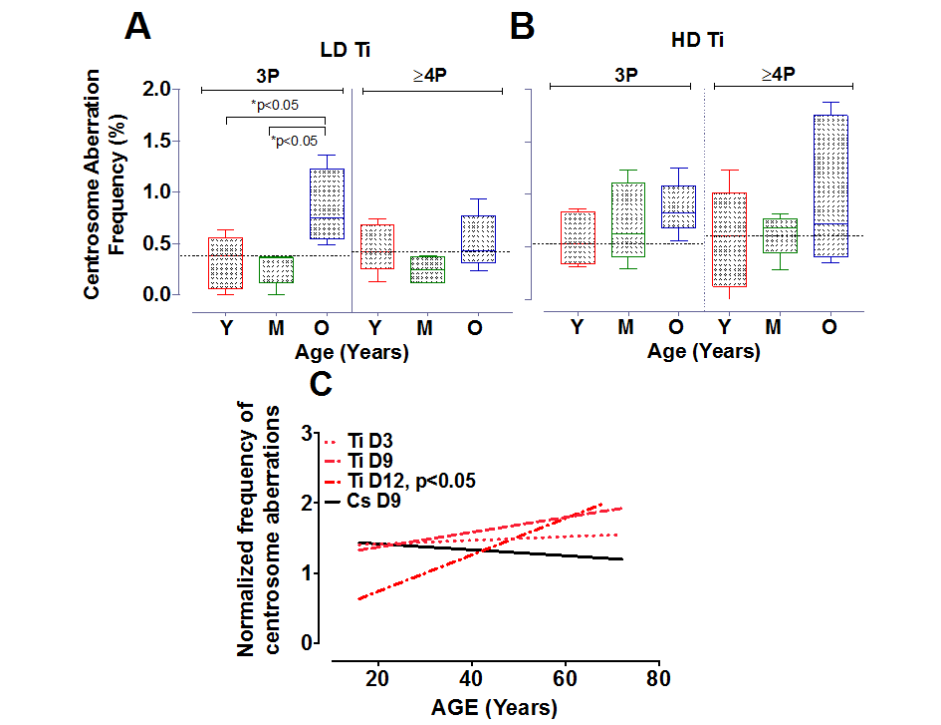
Centrosome aberrations in age-grouped cohorts Strains exposed to Ti at a low dose (LD= 0.05 Gy) or high dose (HD= 0.5 Gy), were sub-grouped into young, middle and old. The frequency of cells with 3P and ≥4P was compared between the different age ranges (**A** and **B** respectively). The horizontal black dotted line represents the mean frequency of centrosome aberrations in the younger age groups. Error bars represent maximum and minimum values within each age group. (**C**) Centrosome aberrations were assessed at 3 days (D3), 9 days (D9) and 12 days (D12) post-exposure. Regression lines that model the relationship between age and the normalized mean centrosome aberration frequency are plotted for the various days. Data represent two independent sets for D3 and D12 for Ti ion and D9 for CS. Data from 4 independent experiments are plotted for D9 Ti.

To further investigate the relationship between the number of aberrant centrosomes per cell and age, data was grouped into three age ranges, namely young (16-30 yo), middle (31-50 yo) and old (51-72 yo). The mean fold change in 3P and ≥4P frequency with Ti exposure was plotted for all three aged cohorts (Fig. 3). With low dose Ti exposure, we observed a significant increase in the 3P population in the older age group in comparison to both the middle (p<0.05) and younger age group (p<0.05), but no change in the proportion of cells with ≥4P centrosomes (Fig. 3A). The mean increase in 3P/cell for the different sub-groups was 0.86 ± 0.16 for the older subgroup (n=4), 0.27 ± 0.07 for the middle-aged group, (n=5) and 0.32 ± 0.12 for the younger cohorts (n=5). Upon exposure to high dose Ti, the mean frequency of cells containing both 3 and ≥4 centrosomes increases with age, although this increase is not significant (Fig. 3B).

### Time course of generation of centrosome aberrations following radiation exposure

There is strong evidence that centrosome aberrations can cause multipolar spindles that retard mitosis. The inherent growth advantage of normal healthy cells compared to cells containing centrosome aberrations thus poses a serious limitation in assessing frequency of this population at long times post-exposure. Centrosome aberrations have been observed as early as 3 days after radiation exposure [26] and can potentiate further genomic instability in the progeny of radiated cells. To define the time dependence of induction of centrosome aberrations in our aged population, we compared centrosome aberrations elicited by Ti exposure at Day 3 (D3) to those that were evaluated at later time points post-exposure; namely Day 9 (D9) and Day 12 (D12) (Fig. 3C). 4^th^ passage HMEC strains were irradiated with 0.5 Gy of Ti and plated on coverslips one day prior to fixation. Regression lines that model the relationship between age and the normalized mean centrosome aberration frequency (>2P) were plotted for various days. These time course experiments confirm that the numbers of cells with aberrant centrosomes increase as early as D3, but the level of increase relative to control was similar in all age groups. Fitting these data to a linear model showed that the centrosome aberration frequency shows a linear increase with age at D9, as noted in Fig. 2 but is significantly associated with age of the strain at D12 (P<0.05). Data represent an average of 2 experiments for each sample for D3 and D12 and 4 experiments for D9. Only a subset of 8 strains spanning the range of ages was assessed for D3 (16, 19, 21, 49, 51, 66, 68 and 72 years). Two HMEC strains, a 27 y and 72 y old, were not processed for D12 due to insufficient cell numbers. These data reveal that an age dependent increase in centrosome aberrations continues to be observed even ~2 weeks post-exposure. In order to test whether repair signaling is elevated at this time point, we also simultaneously analyzed pATF2 foci on the D12 samples. We noted that the fraction of cells exhibiting aberrant pATF2 foci after Ti exposure decreased with increasing age, though this decrease was not significant (data not shown).

### Radiation exposure increases the number of stem/progenitor cells

**Figure 4:**
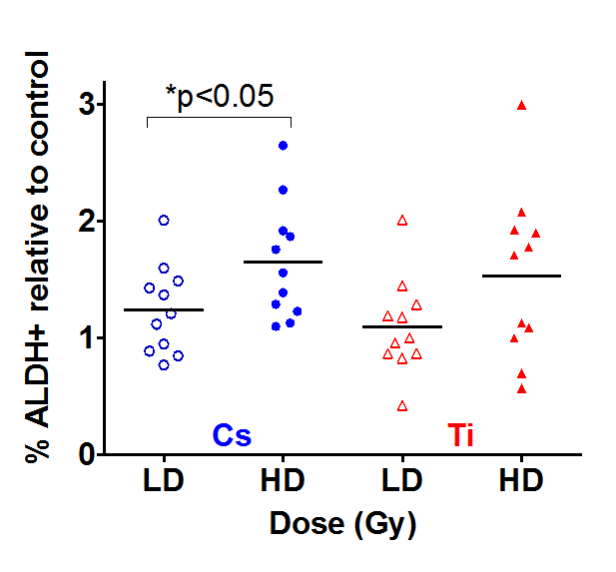
The impact of dose on stem cells numbers Eleven HMEC strains derived from individuals of various ages were exposed to a LD and HD of Cs and Ti ion. Roughly equitoxic doses of Cs and Ti were used for exposures (LD-CS = 0.12 Gy; HD-CS = 0.8 Gy; LD-Ti = 0.05 Gy; HD-Ti = 0.5 Gy). Cells were passaged and processed 9 days after radiation exposure. S/P cells were assessed based on ALDH+ signal using flow cytometry. DAPI negative cells defined the live population. Doublets were eliminated and the ALDH+ signal was assessed with reference to the DEAB control sample. The proportion of ALDH+ cells were plotted relative to the unexposed sham radiated control. Data are based on two independent experiments for high and low dose. Blue and red symbols represent strains exposed to Cs and Ti ion respectively. Empty symbols represent low dose and solid symbols high dose exposures.

Stem/progenitor (S/P) cells have been shown to express higher levels of aldehyde dehydrogenase (ALDH). This population described as ALDH positive (ALDH+) can be used to quantify S/P cell numbers by flow cytometry. Samples were exposed to both a low and high dose of Cs and Ti; subsequently cultured and processed 9 days post-exposure. Single cell suspensions were analyzed for ALDH+ cells to assess radiation-dependent changes in S/P cells. Only eleven HMEC strains were used for ALDH analysis, as we did not have sufficient numbers of viable cells for 4 strains (19, 27, 46 and 72 year old). In analyzing the entire cohort, we noted a radiation-induced increase in the mean proportion of ALDH+ cells relative to control for both types of radiation (Fig. 4). Of interest, this increase was noted even with low dose (5 cGy) exposures. A comparison of the effect of two different doses of Cs indicates that median increase in ALDH+ cells is significantly greater with high dose relative to low dose exposure. A similar although not significant trend was observed with Ti exposure (p=0.08). However, the number of stem/progenitor cells did not significantly differ between exposures that were due to simple as compared to complex damages.

### Age of the cell strain impacts radiation-dependent changes in S/P population

**Figure 5:**
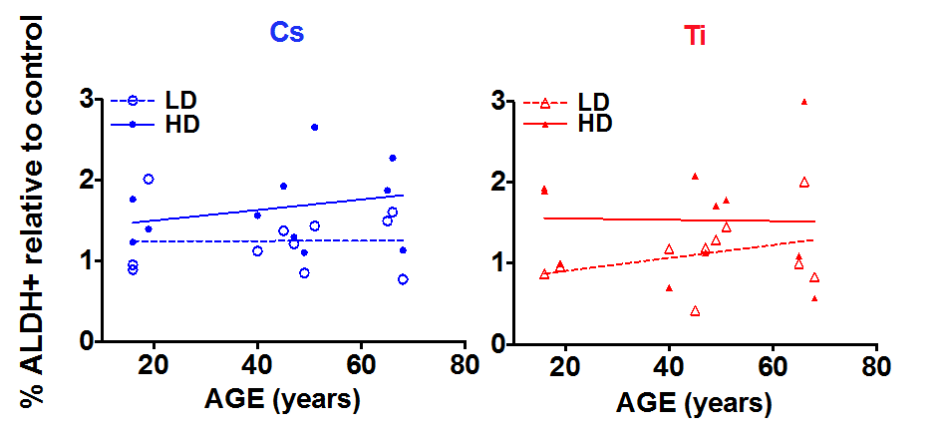
The impact of age on stem cell numbers S/P cells were assessed in HMEC strains based on flow cytometry assessment of ALDH+ signal. The fraction of ALDH+ cells relative to the unexposed control was plotted against age of the individual the strain was derived. Data are based on two independent experiments for high and low dose. Roughly equitoxic doses of Cs and Ti were used for exposures (LD-CS = 0.12 Gy; HD-CS = 0.8 Gy; LD-Ti = 0.05 Gy; HD-Ti = 0.5 Gy). Blue and red symbols represent strains exposed to Cs and Ti ion respectively. Empty circles represent low dose and solid circles high dose exposures. Dotted and solid lines represent the regression lines and note the relationship of stem cell numbers with age for each radiation type.

Regression analysis was carried out to assess the relationship between age and radiation-dependent increase in S/P cells (Fig. 5). While there does not appear to be a correlation between the frequency of ALDH+ cells and age with exposure to low dose Cs, high dose exposure elicits a rising trend with increasing age (Fig. 5A). In contrast, when HMEC strains were exposed to complex damages from Ti exposure, the proportion of ALDH+ cells increased in an age-dependent manner at a low but not a high dose. High dose Ti exposure uniformly increased the proportion of S/P cells in all age groups (Fig. 5B). We further compared dose effects within each age group following exposure to both types of radiation. For the most part, age-grouped analysis confirmed the trends observed with linear regression with one exception. Although there was an age-related increasing trend in S/P numbers with high dose Cs exposure (Fig. 5A), age-grouping indicated that the mean frequency of S/P cells in the older cohort was higher than both the young and middle age groups (Sup. Fig. S2A). In addition, within each aged cohort (young, middle and old), we observed a relative increase in the proportion of ALDH+ population with high dose Cs exposure in comparison to low dose. A similar dose dependent increase has also been noted within each of the three age groups after Ti exposure (Sup. Fig. S2B). Thus an age and dose-dependent increase in numbers of S/P cells is observed following exposure.

### Mechanistic clues that underlie age effects

**Figure 6:**
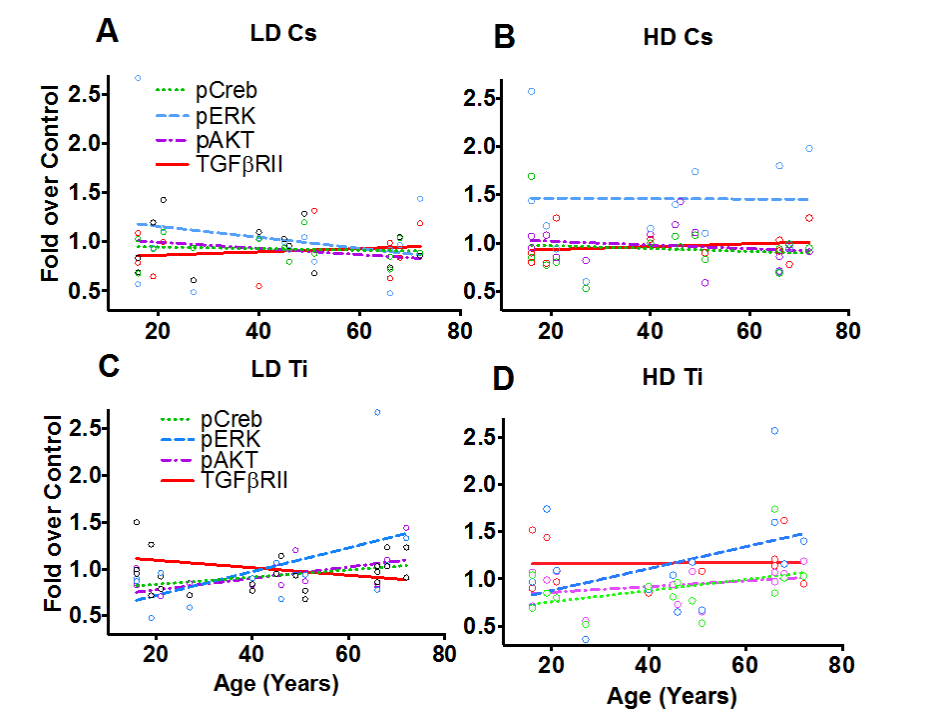
Phospho-protein patterns as a function of age Phospho-profiles of key proteins in the RTK, TNFα and TGFβ signaling pathway were assessed in all fifteen of the HMEC strains 9 days post-exposure to Cs and Ti ions. Roughly equitoxic doses of Cs and Ti were used for exposures (LD-CS = 0.12 Gy; HD-CS = 0.8 Gy; LD-Ti = 0.05 Gy; HD-Ti = 0.5 Gy). Four proteins that showed a change with age were plotted. These include pERK, pCREB, pAKT and total levels of TGFβRII. Dotted lines and solid lines represent regression lines. Trends exhibited with a low (**A**) and a high (**B**) dose of Cs are shown. Similarly, trends observed at a low (**C**) and a high (**D**) dose of Ti ions are shown. Data from two independent experiments were plotted for each radiation type and dose.

Cells activate various signaling networks in response to radiation [27]. These pathways play critical roles in regulating various cell fates, such as cell survival, proliferation, apoptosis, senescence and repopulation and cellular reprogramming. We hypothesized that the cellular response to the radiation would be unique to the nature of damage and show age dependence. To test this hypothesis, samples were processed at 9 days post-exposure to both types of radiation. We assessed the global phosphorylation profile of key nodes in three different stress-activated signaling pathways, namely the receptor tyrosine kinase signaling pathway, TNFα signaling pathway and TGFβ signaling. The fold change in phosphorylation relative to unexposed control was graphed from two independent experiments (Fig. 6). Regression analysis revealed a pronounced age effect in a subset of these proteins that highlights differences in response to simple and complex lesions. While, low dose Cs exposure appeared to show an inverse relationship for phosphorylation of ERK/MAP kinase ½ (Thr^185^/Tyr^187^) with age (Fig. 6A), we noted a uniform increase in phosphorylation with high dose Cs exposure that was independent of age (Fig. 6B). The level of pAKT (Ser^473^), pCREB (Ser^133^) and total TGFβRII protein levels remain unchanged with Cs dose and did not show an association with age (Fig. 6A, 6B). In contrast, in response to complex lesions, we noted a linear relationship between age and the relative increase in level of phosphorylation of ERK/MAP kinase ½ (Thr^185^/Tyr^187^), pAKT (Ser^473^) and pCREB (Ser^133^) following both doses (Fig. 6C). The increase was especially significant for pAKT with low dose exposure (P<0.05). While total protein levels of TGFβRII appear to reciprocally decrease with age at low doses of Ti exposure, we do not observe an association with age at high doses (Fig. 6D). Testing the association between these signaling nodes and previously assessed surrogate markers of cancer risk using Pearson's correlation analysis revealed a significant positive correlation between ALDH+ and centrosome aberrations (P<0.05) and pATF2 and SMAD4 (P<0.05) (Sup. Table S1).

### Phospho-profiles provide information on processes that underlie strain specific variation

**Figure 7:**
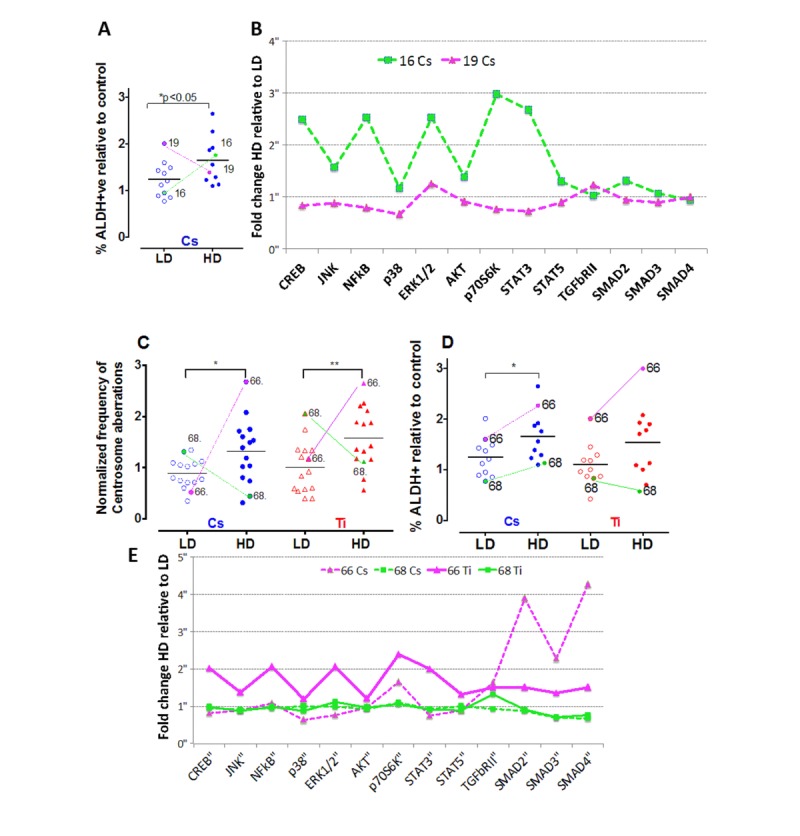
Strain-specific differences in the frequency of centrosome aberrations The fraction of ALDH+ cells were plotted relative to control for cells exposed to low and high doses of Cs (LD = 0.12 Gy; HD = 0.8 Gy) (**A**). Colored lines identify the symbol representing the same strain at low and high dose for two strains of comparable ages (16 y-green and 19 y-pink). Phospho-profiles of key proteins in the RTK, TNFα and TGFβ signaling pathway were compared between the two strains after Cs and Ti exposure (**B**). Fold change of high relative to low dose phospho-protein expression was plotted for the strains. Data represent 2 independent experiments. The fraction of cells with aberrant centrosomes (>3P) (**C**) and ALDH+ cells (**D**) was plotted relative to control for cells exposed to low and high doses of Cs and Ti ion. Roughly equitoxic doses of Cs and Ti were used for exposures (LD-CS = 0.12 Gy; HD-CS = 0.8 Gy; LD-Ti = 0.05 Gy; HD-Ti = 0.5 Gy). Trend lines show that the strains from the 66 y old and 68 y old respond uniquely to low and high doses. Phospho-profiles of key proteins in the RTK, TNFα and TGFβ signaling pathway were compared between the two strains post-exposure to Cs and Ti (**E**). Fold change comparing the high to low dose was plotted for both strains following each radiation exposure (magenta solid line= Ti ion 66 y old strain, magenta dotted line = Cs ion 66 y old, green solid line = Ti ion 68 y old, green dotted line = Cs 68 y old). Data represent two independent experiments for each radiation type.

While a majority of the cell strains showed an increase for centrosome aberrations and stem cell numbers with high dose relative to low dose, for both Cs and Ti exposures, we noted some strain-specific exceptions to this pattern. For stem cell assessments, the strain from the 19 y old showed a dose-dependent decrease with Cs but not Ti exposure (Fig. 7A). In addition, the strain from the 68 y old revealed a dose-dependent decrease in the fraction of ALDH+ cells when exposed to Ti (Fig. 7C), and showed a dose-dependent decrease in the relative frequency of centrosome aberrations generated by both radiation types (Fig. 7D). To examine if protein phosphorylation profiles could provide clues to explain these strain-specific trends, we compared the phosphorylation signatures of these strains to another strain belonging to the same age group. In these strains, we assessed the fold change in the level of the phospho-protein induced by high dose relative to low dose (Fig. 7B, 7E). The data shows that while a HMEC strain from a 19 y old shows levels of pCREB, pNFKβ, pERK1/2, pAKT, p70S6K and pSTAT5 that are notably decreased in the high dose exposed cells relative to the low dose, the strain from the 16 y old shows an increasing trend with dose (Fig. 7B). Contrasting phospho-profiles between the strains from the 66 y old and 68 y old showed that the levels of pCREB, pNFKβ, p38MAPkinase, pERK1/2, pAKT, p70S6K and pSTAT5 are all increased in the 66 y old as compared to the 68 y old following a high dose exposure (Fig. 7E). Interestingly, the levels of pSmad2 (Ser^465^/Ser^467^), pSmad3 (Ser^423^/Ser^425^), as well as total protein levels of TGFβRII and SMAD4 were notably higher in the strain from the 66 y old exposed to low dose Ti relative to high dose Ti compared to inductions in a strain of a woman of similar age (68 y old) (Fig. 7E). These data reveal unique individual expression signatures that change based on dose and lesion complexity.

## DISCUSSION

Genome stability is a major factor that contributes to assessment of cancer susceptibility. Amongst different age groups, the mechanisms that disrupt genomic stability and initiate and/or promote a neoplastic phenotype could vary in nature and/or magnitude based on the type of damage incurred. We used radiation as a tool to provide insight into these processes.

Epidemiological studies in radiation exposed populations suggest that individuals exposed at an early age to simple damages, have an increased cancer risk [5]. Our goal was to understand if exposure to complex damages revealed age related susceptibility.

Studies have shown that complex damages overwhelm the cellular repair mechanisms and result in lesions that are either mis-repaired or remain unrepaired. This loss of DNA repair efficacy has been shown to cause genome instability by increasing the mutation load. However, the high incidence of non-clonal genomic instability in the progeny of irradiated cells argues against mutation frequency being the primary driver of radiation carcinogenesis. Increasing evidence suggests that centrosome aberrations could fulfill this role. As aneuploidy is a prominent feature of genomic instability observed in early stages of cancer, the ability of centrosome aberrations to transmit information regarding structural and positional alterations through generations and generate aneuploidy is thought to have important implications for regulation of phenotypes that impact breast cancer development [28]. Our data demonstrates that the frequency of cells with supernumerary centrosomes is higher following exposure to an agent that primarily produces complex damages in comparison to one that produces mainly simple lesions. High dose exposures significantly increase the aberration frequency for both types of radiation. These results are in line with previous data that have shown a dose-dependent increase in centrosome aberrations with exposure to simple and complex damages [25, 26]. However, a linear relationship between the frequency of radiation-induced centrosome aberrations with age of the exposed cell strain was exclusively noted only with exposures to a radiation type that caused complex damages. These studies reveal that high dose exposure to complex damages generates more cells containing higher numbers of centrosomes (>4) per cell relative to control. There is also a notable statistical correlation between the age of the strain and the fraction of aberrant cells with four or more centrosomes.

What could be causing the increase in centrosome numbers in the older population? Centrosomes typically duplicate once per cell cycle in a semi conservative fashion starting at the G1/S boundary and ending in the G2 phase, prior to mitosis [29]. The higher number of centrosomes per aberrant cell in the older population exposed to complex damages could be attributed to deregulation of the centrosome duplication cycle caused by prolonged cell cycle arrest (sustained G2/M) block [30]. Age related changes in the nuclear lamins and nuclear pore complexes are also thought to alter chromatin organization and affect nuclear integrity. This in combination with decline in repair capacity with age could result in poor DNA repair fidelity further prolonging cell cycle arrest. In our experiments, the fewer population doublings in the older cohort (average PD~1), in comparison to the younger cohort (average PD~2.5) post-exposure to complex damages suggests a more prolonged cell cycle block likely caused by the well-documented decline in repair capacity with aging. Alternatively, there is evidence pointing to increased fragility of the centrosome complex with radiation exposure [31]. Thus, fragmentation could be an additional mechanism for generating supernumerary centrosomes in the older strains. Radiation has also been shown to disintegrate the integral structure and impact the microtubule nucleation ability of centrosomes [31], both notable features implicated in loss of tissue architecture in high-grade breast tumors [32, 33]. The impact of complex damage and age on the fragility of the centrosome complex and its function is not known and would need to be further characterized to provide a comprehensive picture of the effect of radiation on centrosome biology. Nevertheless, a sum of these effects could contribute to the increased sensitivity observed in older strains.

The magnitude of the age-related increase in centrosome aberrations with high doses of Ti highlights dose effects. We noted an approximate 1.8-fold rise in the frequency of centrosome aberrations with a 10-fold increase in dose, revealing centrosome aberration induction following complex damages elicited by Ti not proportional to dose. This lack of linearity suggests that the centrosome aberration frequency is not proportional to the initial damage but instead could reflect chronic phenotypes that persist weeks post-exposure. One likely candidate is chronic oxidative stress caused by ROS that are both endogenously generated as a product of cellular metabolism or by exposure to factors such a radiation [34]. These free radicals and their reactive oxidant intermediates have been implicated in causing deleterious changes in DNA, lipids and protein [35]. A delicate balance between the concentration of ROS and antioxidant species is essential for normal cellular homeostasis. However, loss of this equilibrium can result in a pro-oxidative state, termed as oxidative stress [36]. In older individuals, a combination of increased ROS and impaired anti-oxidative redox enzymes such as catalase, super oxide dismutase and glutathione peroxidase are thought to contribute to age related deterioration of function [37-39]. Studies have shown that complex damages elicit a higher degree of persistent oxidative stress in comparison to simple lesions [40, 41]. This increase is a heritable non-targeted effect that has been noted in the progeny of irradiated cells [42]. Irradiation of aged cells could further exacerbate the existing pro-oxidative phenotype in the older population by overwhelming the ability of antioxidant systems to neutralize ROS [39, 43]. This cumulative increase in oxidative burden in the older population could be one of the causative factors for higher centrosome aberration frequency in older cells exposed to high doses of complex damage. Although we have noted a clear age effect by regression analysis, strain specific variation in biological response influences the significance of trends in age grouped analysis (n=5). Further studies with increased sample sizes for each age group would be useful to validate the observed trends.

We also evaluated stem cell frequency in relation to age and radiation exposure. Our studies show that S/P cell numbers increase with dose for both radiation qualities. This is consistent with previous studies, which have shown that a fraction of S/P cells increase with radiation exposure [21, 44]. Similar to centrosome aberrations, the radiation induced increase in the S/P population was also not proportional to dose, however in contrast, a universal age dependent effect is not seen. Rather only at a low dose of Ti and a high dose of Cs is a slight age-dependent effect observed. At a higher dose of Ti a universal high increase in stem/progenitor cell numbers is seen, whereas at a low dose of Cs all age groups show no difference from controls. Thus a slight increase with age is only observed at intermediate levels of damage. It is likely that the mechanism underlying elevation in the stem cell numbers could differ between the different aged groups. Increases in stem cells with radiation could be attributed to 1) increased survival based on known inherent radio-resistance of this population; (2) increased self-renewal of stem cells to repair and repopulate the niche in order to counter act the effects of damage; or (3) reprogramming of terminally differentiated epithelial cells by radiation exposure, causing them to dedifferentiate into a S/P cell type. Recent work by Guo and coworkers present an alternative mechanism of reprogramming that could underlie the increase in S/P cells we observe in the older population. They suggest that pre-senescent HMECs such as those induced by radiation, can undergo phenotypic reprogramming to generate more stem cells [45]. We have noted that older cells have fewer population doublings following Ti (average PD~1) relative to Cs exposure (average PD~2.5). As apoptosis is not generally observed with the doses used in this study, it is likely that radiation induced senescence impacts cells in the older population leading to fewer population doublings. This possibility is supported by our previous work that showed a radiation induced increase in the proportion of cells that stain positive for beta-galactosidase, a known marker for senescence (data not shown). Given this, it is intriguing to speculate that reprogramming of a higher proportion of senescent cells in older strains could be a viable mechanism at play in generating S/P cells from this population following radiation exposure.

While our results reveal that the relative fraction of S/P cells does not universally increase with age following all dose and radiation quality exposures, we cannot exclude the possibility that their ability to differentiate is impacted by the complexity of the lesion induced. Aging has been associated with increased stem cell dysfunction. Under conditions of stress, while a fraction of progenitors decrease due to increased apoptosis with stress, the remaining stem cells are thought to leave their quiescent stage and divide more rapidly to undergo self-renewal and expansion to compensate for the cell loss and prevent premature exhaustion of the stem cell niche [46]. As both surrogate markers of cancer were assessed independently, we do not know whether centrosome aberration frequency and by inference genomic instability in the stem cell population will exhibit a similar linear relationship with age in response to complex damages. Observation of these alterations within weeks post-exposure suggests that these changes point to processes that promotes rather than initiates carcinogenesis. As radiation causes an increase in stem cells, it is not unreasonable to expect that the higher levels of DNA damage and genomic instability induced by this exposure could impact their function to differentiate into various cell types. As stem cell dysfunction can directly impact tissue composition and by extension integrity and architecture, it is clearly important to further elucidate the function of stem cells in the context of age and lesion complexity.

Assessment of global phospho-protein signatures has provided some clues as to the possible signaling mechanisms that underlie the age-dependent effects we observed. They also reveal signaling can influence strain specific differences in biological response. Further characterization of these pathways in isolated S/P cells would be essential to better understand how aberrations in these cells could foster cellular transformation.

**Figure 8:**
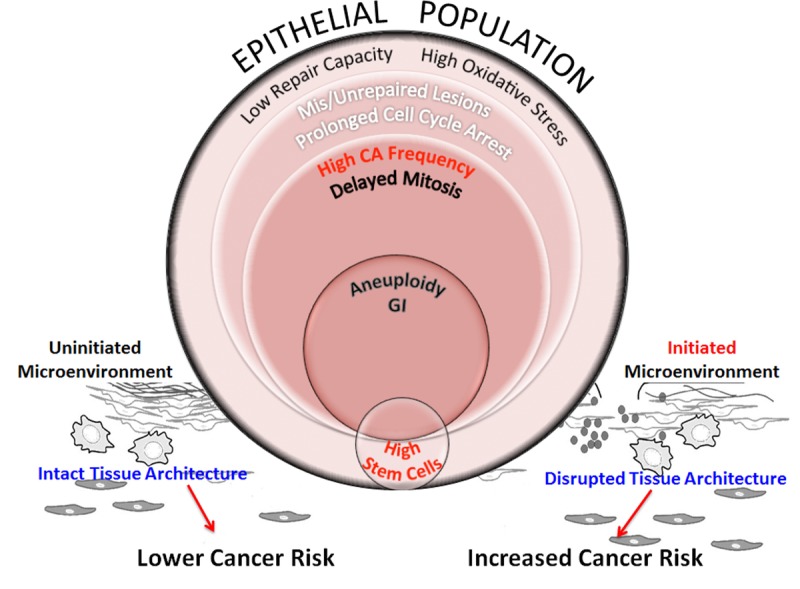
Schematic representation of processes that increase genomic instability in epithelial cells from older individuals exposed to complex damage The model hypothesizes the interplay between processes that potentially alter cancer risk in older women exposed to complex lesions. Exposure of HMECs from older individuals to complex damages initiates a cascade of interconnected events. Poor repair capacity and increased oxidative burden in the older cohort impairs repair fidelity. Impaired redox balance caused by complex damages could further hamper lesion repair resulting in a fraction of cells with unrepaired and mis-repaired lesions resulting in prolonged cell cycle arrest. At the cellular level, centrosome amplification can result in the formation of multipolar spindles that slow down mitosis and cause mitotic abnormalities. While some of these cells are targeted for mitotic cell death, apoptosis or senescence, other cells exhibit aneuploidy. When these genome-destabilizing events occur in stem cells, they could cause catastrophic results by increasing genomic instability in the progeny. Although we don't observe a significant linear relationship between radiation-induced stem cells with increasing age (Fig 5B), the higher centrosome aberration frequency in older cells (Fig 3B), increases the potential for genomic instability within this population. This will likely impact their ability to differentiate into various cell types thus altering tissue composition and impairing the structural integrity of the tissue. However, the proliferative advantage required for propagation of this genomic instability in stem cells would require additional cues from the microenvironment. Senescent cells, activated stroma and inflammation exhibit an age-dependent incidence due to non-targeted effects of radiation. These events are key candidates in the tissue microenvironment that provide the stimuli to either promote or inhibit carcinogenesis. An uninitiated microenvironment can confer protective effects by restricting promotion of cancer phenotypes and maintaining normal tissue architecture. However, an initiated microenvironment can create a permissive milieu for epithelial carcinogenesis by augmenting genomic instability and disrupting tissue architecture. We postulate that risk would be defined by a complex interplay between all of these factors including repair capability, oxidative stress, genomic instability, stem cell changes, and the integrity of the tissue architecture.

The etiology of breast cancer involves a complex interplay amongst various factors such as repair capability, oxidative stress, genomic instability, stem cell effects and integrity of tissue architecture that contribute to susceptibility in all age groups. We postulate that when HMECs from older individuals are exposed to complex damages, this exposure unfolds a cascade of interconnected events (Fig. 8). Poor repair capacity in the older cohort impairs repair fidelity. In older cells, impaired redox balance caused by complex damages may further hamper lesion repair by enhancing protein oxidation and nuclear disorganization. This results in cells with unrepaired and mis-repaired lesions that cause cell cycle arrest. In a fraction of cells with either prolonged cell cycle arrest or fragile centrosomes or both, the centrosome cycle is deregulated resulting in the generation of supernumerary centrosomes. As oxidative stress has also been shown to contribute to the generation of centrosome aberrations, the pro-oxidant state in older people is likely to enhance centrosome aberration frequency and further potentiate genomic instability [47-50]. At the cellular level, centrosome amplification can result in the formation of multipolar spindles that slow down mitosis and cause mitotic abnormalities. Cells have inherent mechanisms to protect against transformation by inducing mitotic cell death, apoptosis or senescence. However when these genome-destabilizing events occur in stem cells, they could cause catastrophic results by increasing proliferation of this unstable stem cell population while simultaneously allowing them to evade apoptosis, both of which are intrinsic features in carcinogenesis [51]. Although we don't observe a significant linear association between a radiation-induced increase in stem cells and age, we do see a slight age effect when comparing grouped ages at intermediate levels of damage (Sup. Fig. S2; comparing high dose of Cs or a low dose of Ti). Pearson's correlation analysis has also revealed a strong association between ALDH+ signal and centrosome aberration frequencies (Sup. Table S1). The higher levels of centrosome aberrations and levels of genomic instability in older cell strains suggest these stem cell populations may be at a greater risk for mutation. This increased mutational load will likely impact their ability to differentiate into various cell types thus altering composition and impairing the structural integrity of the tissue.

Conventional view suggests that aneuploidy caused by centrosome aberrations could be the predominant causal factor in increasing genomic instability within stem cells in the older population [30]. However evidence of an important role for the centrosome in regulating asymmetric division of stem cells opens up another intriguing possibility for generating genomic instability in older individuals [52]. If proven, the fact that abnormal centrosomes can drive malignant transformation by deregulating asymmetric cell division, an integral property of stem cells, could cause a paradigm shift. Increased centrosome amplification in aged and oxidatively stressed stem cells in the drosophila mid-gut and its link to polyploidy suggests the mechanism of propagating genome instability in older cells could be by modifying stem cell division in a centrosome dependent manner [53].

Lastly, though we have centered our analysis on epithelial cells, in the broader context, we cannot ignore the fact that the neoplastic phenotype is context dependent. While centrosome aberrations can destabilize the genome of stem cells, the proliferative advantage required for propagation of this genomic instability requires additional impetus. Senescence, stromal effects and inflammation are key candidates that can provide this stimulus and are known to exhibit age related changes. Although senescence is part of the cellular arsenal to maintain genomic integrity, groundbreaking studies by the Campisi group have shown that senescence accompanied by the SASP phenotype can prime transformation of genomically unstable cells [54, 55]. Thus, increased senescence typically observed in tissues of the older population could further promote transformation. Interestingly, while increased centrosome aberrations are seen in prematurely senescent cells, it is not clear, if this is a cause or consequence of senescence [56]. There is mounting evidence that stromal-epithelial interactions play a crucial role in breast carcinogenesis. Stroma has been shown to prevent neoplastic growth by reprogramming neoplastic epithelial cells to function normally [57]. Evidence of spontaneous regression of clinical lesions in older people are thought to be attributed to the protective effects of the stroma [58]. However, a wealth of data reviewed by Barcellos-Hoff et al, have shown that radiation can alter the signaling within the stroma and promote a microenvironment that is more permissive to carcinogenesis [59]. Likewise inflammation is another common feature between aging and radiation that sets the context for carcinogenesis. A summary of pathology reports of the reduction mammoplasty samples indicates mild fibrocystic disease in many of the HMEC strains over the age of 40 (data not shown). It is uncertain how complex damage contributes to the existing level of inflammation, but future studies looking at the combined effects of senescence, stroma and inflammation will aid in completing this picture. Recent modeling of cancer risk in the context of age of exposure of A bomb survivors suggests that while risk in younger individuals is dominated by the cancer initiation, promotion of premalignant cells that normally exist in individuals, and likely increase with age, has a major contribution in older individuals [60]. Given this, we can speculate that increased genomic instability within the stem cell population of older people, especially post-exposure to complex damages could potentially place them at higher cancer risk, as promotion of existing initiated cells could be higher with heavy ion exposure. Additional studies more fully investigating this conjecture would be required to validate this hypothesis.

In conclusion, to our knowledge this is the first report revealing an age related increase in genomic instability with increasing dose and lesion complexity in normal primary HMECs. Given stem cell numbers are increasing with radiation, the age-dependent global increase in genomic instability could also impact the S/P cell population. However, potentiation of this genomic instability will be subject to other non-targeted effects such as senescence, stroma and inflammation. Further investigation of the interplay between these factors is necessary to better define cancer risk from these exposures in the context of aging.

## MATERIALS AND METHODS

### Cell strains

We have chosen fifteen cell strains from the Human Mammary Epithelial Cell Bank at LBNL to test from a cohort of well-characterized pre-stasis finite-lifespan normal human mammary epithelial cells (HMEC) derived from a wide range of aged individuals. These HMEC strains were produced in Dr. Martha Stampfer's laboratory at LBNL, Berkeley CA, and originated from a large bank of uncultured organoids derived from reduction mammoplasty of normal breast tissue. Details on the derivation and culture of these HMECs can be found on http://hmec.lbl.gov**.** For some analyses we have classified strains into three age-defined cohorts based on the age of the individual from which the strain was derived. Four to five strains were chosen from various age ranges. The young cohort contained strains from women 16-27 years old (16, 16, 19, 27, 30 yrs), the middle-aged cohort contained strains from women 40 to 49 years old (40, 45, 46, 47, 49 yrs) and the old cohort contained strains from women 51-72 years old (51, 65, 66, 68, 72 yrs). These strains were previously used as part of a wider cohort in a study by Garbe et al who reported age dependent defects in the functional differentiation of multi potent progenitors [23].

### Cell culture

Passage 2 (P2) cells were cultured and passaged to generate adequate P4 cell stocks for all experiments. These early passage (P4) mammary strains were grown to sub-confluence (~85%) in T75 flasks and exposed to radiation. Cell strains were maintained in serum-free M87A medium supplemented with 0.5 ng/ml cholera toxin (CT), 0.1 nM oxytoxin (Bachem) and 1X antibiotic-antimycotic (Invitrogen) [61]. Cultures were fed every two days. Cells were routinely tested for absence of mycoplasma contamination (Bionique Test Labs, NY) prior to use.

### Irradiation

Strains plated in T25 flasks were exposed to two different radiation qualities at room temperature. Heavy ion exposures with Ti 300 MeV/n (nucleon) were carried out at the NASA Space Radiation Laboratory (NSRL) at Brookhaven National Laboratory, Upton, NY. To prevent particle fragmentation, cell culture flasks were placed perpendicular to the beam and oriented with the cell monolayer side of the flask facing the beam. Two to four independent experiments were carried out over the course of a two-year period at two different NSRL campaigns (NSRL14C and NSRL15B). At the NSRL14C campaign cells were exposed to just one dose (0.5 Gy), and centrosome aberrations were assessed at three different time points; day 3, day 9 and day 12 post-exposure. In the subsequent NSRL15B campaign, to assess low dose effects, we included a low dose (0.05 Gy) along with the high dose exposure and processed samples only at day 9. Dose rates ranging from (5.5-6.4) cGy/min for low dose exposures and (22.23-32.5) cGy/min for high dose exposures were used to maintain short exposure times (0.5-2.0 minutes). Cells were grown for 24 h following irradiation and either shipped back to LBNL as live cultures or frozen down at BNL for analysis at a later date. Upon arrival at LBNL, cells were allowed to acclimatize for a day and subsequently trypsinized, viability checked and reseeded at low concentrations in either 60 mm dishes or in 100 mm dishes for long-term culture to subsequently define centrosome analysis or to assess stem cell numbers. Cells were exposed to Cesium radiation at Lawrence Livermore National Laboratory at dose rates of ~25 cGy/min for low doses and ~100 cGy/min for high doses and processed similar to the Ti samples. Previous data, evaluating double strand break induction using ϒH2AX foci in a primary HMEC strain was used to calculate Cs doses that generate the same amount of initial damage as Ti (unpublished results). Based on these results, dose delivered with Cs exposures were ~1.5 fold higher than Ti for both the low and high doses to compare endpoints in response to approximately equitoxic doses. Two independent experiments were carried out for cesium exposures. Total population doublings for each culture was calculated beginning at passage 5 prior to the radiation exposure, using the formula PD= log_2_ (recovered viable cell number/number of viable cells seeded)/0.301. Population doublings ranged from ~1-6, the proliferative potential of these strains is 10-15 PDs (personal communication J. Garbe).

### Centrosome aberrations

For centrosome aberration analysis, cells were trypsinized 48 h after radiation exposure, and seeded at low density in 60mm dishes. In NSRL 14C campaign, Ti exposed cells were trypsinized on D2, and D8 and re-plated onto coverslips to analyze samples at D3, D9 and D12 after radiation exposure. Cesium samples were analyzed only at D9 post-exposure to compare with D9 Ti exposed samples. Coverslips were fixed in 100% methanol for 10 min at −20^o^C, washed in PBS and subsequently stained for pericentrin. Coverslips were blocked in 1% BSA for 1 h at RT and centrosomes were detected by indirect immunofluorescence, using a primary rabbit antibody against pericentrin (1:1000) (Abcam, Cambridge MA). Following primary antibody incubation (1 h at RT), the cells were washed three times in blocking buffer, and subsequently incubated for 1 h with goat anti-rabbit Alexa Fluor 594 (Invitrogen, Waltham MA). Following secondary antibody incubation, cover slips were washed twice in PBS, counterstained with 0.1 μg/ml 4′, 6-diamidino-2-phenylindole (DAPI) in PBS, air-dried and mounted with Vectashield (Vector Laboratories, Burlingame, CA). Stained cells were imaged with a Zeiss Axiovert 200 M inverted fluorescence microscope equipped with a cooled CCD camera and Image-Pro®Plus software (MediaCybernetics, Rockville MD). Image acquisition was carried out within a week of staining.

### Calculation of centrosome aberrations

Centrosome aberrations were scored blind and at representative areas throughout the slide. For each region we assessed the total number of cells based on DAPI stain, and the number of cells with aberrant centrosomes (>2). Images of cells with aberrant numbers of centrosomes were taken at 40 X and scored by two independent observers to evaluate the number of centrosomes in each aberrant cell. The type of centrosome aberration was characterized as either 3 centrosomes/cell (3P) or greater than 3 centrosomes/cell (>4P). Between 200-400 cells were examined for each sample.

### Assessment of ploidy via flow cytometry

1x10^5^ cells from samples were fixed with 100% methanol, washed in PBS and subsequently stained with propidium iodide (PI). Ploidy was determined by flow cytometry based on DNA content and PI staining. DNA content of 10,000 cells was assessed on the FL2 channel using a BD FACS Calibur. Data was analyzed using Flowjo software Ver 8.6 (Tree Star Inc. OR).

### Assessment of stem cell numbers

For assessing the proportion of stem cells we have used ALDEFLUOR reagent (STEMCELL Technologies), which has been shown to be specific for stem/progenitor cells in mammary epithelial cell populations. A previous study [62] using a derivative of one of our strains (184A1), revealed that pre-sorting cells using the ALDEFLUOR kit improves mammosphere generation by ~3 fold. Radiated and sham exposed cell strains were grown in 100 mm dishes and trypsinized 9 days following radiation exposure. Single cells in suspension were filtered using a 35 μ nylon mesh, counted using a hemocytometer and set up at the suggested concentrations for ALDEFLUOR labeling as per established protocols (STEMCELL technologies Inc., Vancouver). DAPI was included in the staining protocol for exclusion of dead cells that stain positive for DAPI (DAPI+). Cells expressing high levels of aldehyde dehydrogenase activity (ALDH+) were identified by their bright green fluorescence and enumerated from the total mammary cell population on a BD FACS Vantage SE flow sorter based on the FL1 channel. Gates for the ALDH+ population were defined using the supplied negative control that includes DEAB, an inhibitor of ALDH activity. Data are presented as proportion of stem cells in the radiated samples relative to sham-irradiated controls.

### pATF2 immunostaining

For analysis of persistent pATF2 foci, coverslips processed on D12 following Ti exposure were co-stained with a mouse monoclonal antibody specific for pericentrin (Abcam, Cambridge MA) and a rabbit polyclonal antibody against pATF2 (Ser^490/498^) (1:1000) (Rockland Inc, Gilbertsville PA). Following primary antibody incubation (1 h at RT), the cells were washed three times in blocking buffer, and subsequently incubated for 1 h with goat anti-mouse Alexa Fluor 594 (Invitrogen, Waltham MA) and goat anti-rabbit Alexa Fluor 488 (Invitrogen, Waltham MA). Following secondary antibody incubation, cells were washed twice in PBS, counterstained with 0.1 μg/ml 4′, 6-diamidino-2-phenylindole (DAPI) in PBS, air-dried and mounted with Vectashield (Vector Laboratories, Burlingame). Image acquisition was carried out within a week of staining. Data from two independent experiments were analyzed. Cells with >3 pATF2 foci were stratified as aberrant cells exhibiting persistent pATF2 signal. 200 cells were scored for each sample.

### Multiplex analysis for phospho-protein expression

Two sets of samples were exposed to Ti (NSRL15B, BNL) and Cs (LLNL13A, 14C, 15B). Both irradiated and sham samples were trypsinized and grown in 100 mm dishes and processed 9 days post-plating. Six plates were processed in each batch. After a wash with cold PBS, 300 ul of ice-cold 1X Milliplex lysis buffer with protease inhibitors (Sigma) and phosphatase inhibitors (1 mM sodium orthovanadate, Sigma) was added to each plate on ice. Cells were scraped off the plate, and cell suspensions were transferred to a centrifuge tube and rocked for 15 mins at 4^o^C. Particulates were removed by high-speed centrifugation 12000 rpm for 10 mins at 4C, and protein concentration was determined by the BCA assay on the nano-drop. Samples were aliquoted and stored at −70^o^C. The MILLIPLEX® MAP 9-plex Multi-Pathway magnetic bead signaling kit was used to detect the phosphorylation profiles of key proteins in two different stress activated signaling pathways, namely the receptor tyrosine kinase (RTK) signaling pathway and TNF alpha signaling pathway. This kit was used to detect changes in phosphorylated ERK/MAP kinase ½ (Thr185/Tyr187), AKT (Ser473), STAT3 (Ser727), JNK (Thr183/Tyr185), p70 S6 kinase (Thr412), NF-κB (Ser536), STAT5A/B (Tyr694/699), CREB (Ser133), and p38 (Thr180/Tyr182) in cell lysates using the Lumina® system. The MILLIPLEX MAP Human TGFβ Signaling Magnetic Bead Panel 6-plex, was used to detect changes in phosphorylated Smad2 (Ser465/Ser467), Smad3 (Ser423/Ser425), as well as total protein levels of TGFRβII and Smad4 in cell lysates using the Luminex® system. 25 ugs of sample was used in each well and the immunoassay was carried out according to the published protocol for this kit (48-680 MAG, Millipore) and analyzed using the Luminex® system.

### Statistical analysis

Linear regression was used to determine trends in surrogate endpoints for cancer relative to age. One-way ANOVA were used for multiple comparisons. Statistical significance of data was determined with p values <0.05. Pair wise comparison of differences in the percentage of cells with centrosome aberrations among dosed and unexposed controls were carried out using the Wilcoxon test. Differences in centrosome aberration frequency and stem cells amongst age-grouped cohorts were analyzed by the t test for independent samples. Pearson's correlation was used to examine the correlation between frequency of centrosome aberrations, ALDH+, pATF2 persistent signal and the relationship of these parameters to the level of various phospho-proteins in the stress activated signaling pathways. Analysis of variance (ANOVA) was used to estimate the 95% confidence interval of the mean. All statistical analysis was performed using GraphPad Prism 6.0.

Research was conducted under Lawrence Berkeley National Laboratory Human Subjects Committee IRB protocols 305H002 and 108H004.

## SUPPLEMENTAL MATERIAL FIGURES AND TABLE



## Abbreviations

HMEC: Human mammary epithelial cells
ALDH: Aldehyde dehydrogenase
ROS: Reactive oxygen species
LD: Low dose
HD: High dose
S/P: Stem/Progenitor
Cs: Cesium
Ti: Titanium
RTK: Receptor Tyrosine Kinase
