# Supplementary material for: Lesion complexity drives age related cancer susceptibility in human mammary epithelial cells
Source: Aging (Albany NY). 2017 Feb 28;9(3):665–84. doi: 10.18632/aging.101183 (PMC5391225; doi:10.18632/aging.101183)
Supplement: Supplementary file 1 [file aging-09-665-s001.pdf]

SUPPLEMENTARY MATERIAL

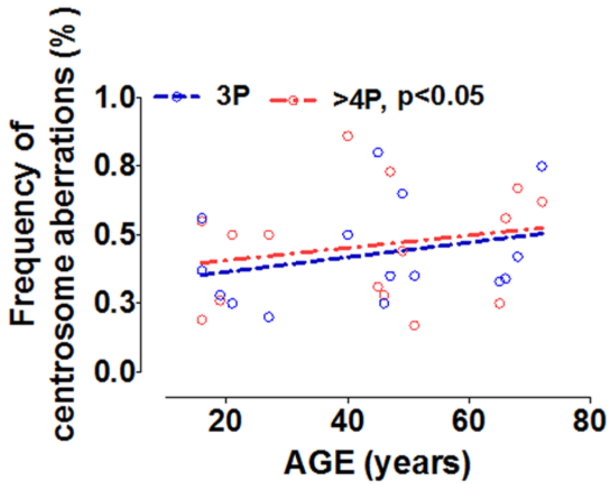

Supplementary Figure S1. Centrosome aberrations in control samples. The fraction of cells with 3 pericentrin foci (3P) and ≥4 pericentrin foci (≥4) were plotted in unexposed cells for all strains as a function of age. Regression analysis was carried out to define the relationship between the frequency of cells with centrosome aberrations and age in unirradiated cells.

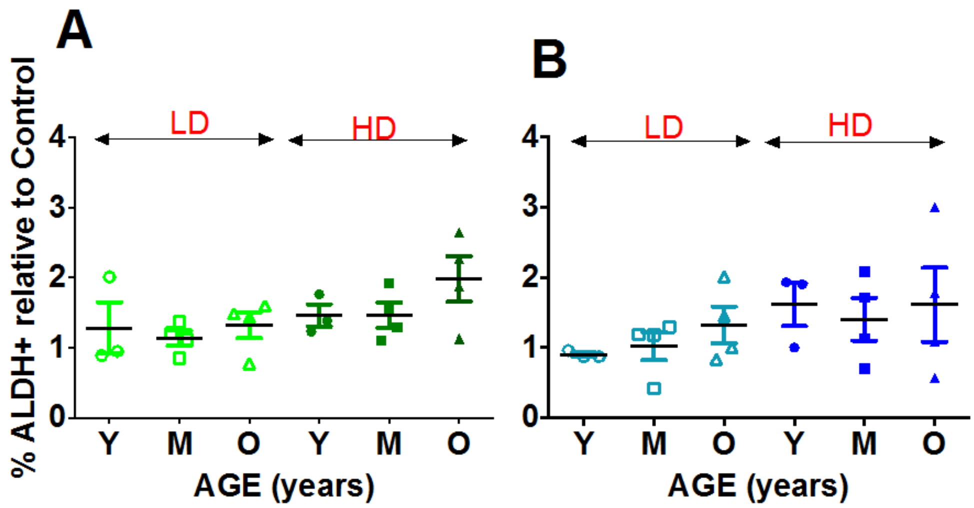

Supplementary Figure S2. Comparison of stem cell numbers in strains of various age groups. S/P cells were assessed in HMEC strains based on flow cytometry assessment of ALDH+ signal. The fraction of ALDH+ cells relative to the unexposed control was assessed. Strains were sub-grouped into young, middle and old represented by circles, rectangles and triangles, respectively. Filled symbols represent the high dose and open symbol represent the low dose. The frequency of S/P cells relative to control was compared between the different groups. Error bars represent SD within each age group. Data are based on two independent experiments for high and low dose. Data from both Cesium exposure (**A**) and Ti ion exposure (**B**) are presented. A slight age-dependent increase in S/P cell populations is noted with a low dose of Ti and a high dose of Cs.

Supplementary Table S1. Correlations between various endpoints.

Pearson's correlation analysis

Markers	CA	ALDH+	pATF2 ($Ser^{490/498}$)
CA		+ ($p<0.05$)	-
ALDH+	+ ($p<0.05$)		-
pATF2 ($Ser^{490/498}$)	-	-	
p38 (Thr^{180}/Tyr^{182})	+	-	+
p70 S6K (Thr^{412})	+	-	+
pAKT(Ser^{473})	+	-	+
pCREB(Ser^{133})	+	-	+
pERK/MAPK(Thr^{185}/Tyr^{187})	+	-	+
pJNK (Thr^{183}/Tyr^{185})	-	-	+
pNFκβ (Ser^{536})	-	-	+
pSTAT3(Ser^{727})	-	-	+
pSTAT5 A/B ($Tyr^{694/699}$)	-	-	+
TGFRβII	+	+	+
pSMAD2 (Ser^{465}/Ser^{467})	+	+	+
pSMAD3 (Ser^{423}/Ser^{425})	-	-	+
SMAD4	+	+	+ ($p<0.05$)

Pearson's correlation was used to examine the association between centrosome aberration (CA) frequency, ALDH+ signal, persistent pATF2 foci, and the relationship of these parameters to the levels of various phospho-proteins in the stress activated signaling pathways.
